# Interactive Effects of *Epichloë* Endophytes and Arbuscular Mycorrhizal Fungi on Saline-Alkali Stress Tolerance in Tall Fescue

**DOI:** 10.3389/fmicb.2022.855890

**Published:** 2022-04-25

**Authors:** Hui Liu, Huimin Tang, Xiaozhen Ni, Yajie Zhang, Yingchao Wang

**Affiliations:** College of Life Sciences, Dezhou University, Dezhou, China

**Keywords:** endophyte, arbuscular mycorrhizal fungi, interaction, tall fescue, saline-alkali stress

## Abstract

*Epichloë* endophytes and arbuscular mycorrhizal fungi (AMFs) are two important symbiotic microorganisms of tall fescue (*Lolium arundinaceum*). Our research explores the combined effects of endophytes and AMF on saline-alkali stress. The finding revealed that a significant interaction between *Epichloë* endophytes and AMF, and saline-alkali stress occurred in the growth and physiological parameters of tall fescue. Endophyte infection significantly enhanced tall fescue resistance to saline-alkali stress by increasing shoot and root biomass and nutrient uptake (organic carbon, total nitrogen, and total phosphorus concentration), and accumulating K^+^ while decreasing Na^+^ concentration. Furthermore, the beneficial effect of endophytes was enhanced by the beneficial AMF, *Claroideoglomus etunicatum* (CE) but was reduced by the detrimental AMF, *Funneliformis mosseae* (FM). Our findings highlight the importance of interactions among multiple microorganisms for plant performance under saline-alkali stress.

## Introduction

*Epichloë* endophytes [Clavicipitaceae, Hypocreales, and Ascomycota] are endophytic fungi that can infect and coexist with above-ground portions of host plants and have been shown to impact their tolerance to certain biotic and abiotic stresses such as drought, low nitrogen, salt, cold, heat, heavy metals, insects, nematodes, and diseases by either enhancing the fitness and productivity of host plants or producing a range of alkaloids and other secondary metabolites (Schardl et al., 2004; Wang et al., 2021). In recent years, there has been a lot of research interest in the salt tolerance of hosts bearing *Epichloë* endophytes (Rodriguez et al., 2008; Reza and Mirlohi, 2010). Some studies found that under salt stress, endophyte infection enhanced growth, activity of enzymes of nitrogen metabolism, nitrogen use efficiency, and photosynthetic ability (Wang et al., 2019), and that *Hordeum brevisubulatum* infected with *Epichloë bromicola* had greater growth, including improved antioxidant potential, increased nutrient absorption, and osmotic and ionic adjustment (Song et al., 2015; Chen T. X. et al., 2018). Endophytes, on the other hand, may also decrease the salt tolerance of their hosts. For example, Ren et al. (2006) demonstrated that endophyte infection significantly decreased tiller number under high salt stress. Another study indicated that endophytes did not influence the biomass production of *Festuca rubra* under soil salinity conditions (Zabalgogeazcoa et al., 2006).

Plants can also deal with soil environmental stress by interacting with arbuscular mycorrhizal fungi (AMFs), which are members of the Glomeromycota phylum and form mutualistic relationships with more than 80% of terrestrial plant roots belowground and are found in saline and alkaline soils (Aliasgharzadeh et al., 2001; Wilde et al., 2009). Numerous studies have indicated that AMFs play an important role in improving host plant saline-alkaline stress tolerance by improving nutrient and water uptake, maintaining ion balance, increasing photosynthetic efficiency, and inducing the antioxidant defense system (Estrada et al., 2013; Abd-Allaa et al., 2019; Ben-Laouane et al., 2020; Moreira et al., 2020; Yang et al., 2020). In addition, Qiu et al. (2020) found that outcomes of salt stress tolerance of plants varied with AMF species identity. In addition, Parihar et al. (2020) indicated that multi-species AMF inoculation was superior to single AMF inoculation in improving plant production under salt stress conditions.

In grass species, dual infection of a host plant with leaf endophytes and below-ground AMFs is common. Interactions between grass endophytes and AMFs are complex, and studies focused on the two have been inconclusive based on relationships among the plant, AMFs, and grass endophytes. AMF colonization rates could be inhibited (Müller, 2003; Omacini et al., 2006; Mack and Rudgers, 2008) or enhanced (Novas et al., 2005, 2009; Arrieta et al., 2015) by leaf endophytes, and results are dependent on the combination of the AMFs and endophytes as well as soil environmental conditions. Our previous study has discovered that interactions between endophytes and AMFs, as well as their combined effects on growth of host plants depended on both the identity and richness of AMFs (Liu et al., 2019). In addition, Liu et al. (2011) demonstrated that the competition between AMFs and endophytes was determined by resource supply and host carbohydrate content. According to Zhou et al. (2016), tripartite interactions among endophytes, AMFs, and *Achnatherum sibiricum* were influenced by nutrient supply.

Many studies have been conducted to investigate the individual effects of *Epichloë* endophytes and AMFs on the growth of host plants and of the impact of saline-alkaline stress (Song et al., 2015; Chen T. X. et al., 2018; Wang et al., 2019; Moreira et al., 2020; Yang et al., 2020). Research on interactions of the two microorganisms infecting the same host plant is limited, especially regarding saline-alkali stress environments. This study was designed to evaluate the role of *Epichloë* endophytes and AMF species, as well as their interaction effects on the growth of tall fescue (*Lolium arundinaceum*) under saline-alkali stress conditions. As for AMFs, colonization with two AMFs, *Funneliformis mosseae* (FM) and *Claroideoglomus etunicatum* (CE), alone and in combination, as well as non-inoculated (M-), was considered. We analyzed the growth characteristics and physiological parameters of tall fescue. Specifically, we addressed the following questions: (1) does endophyte infection ameliorate saline-alkali stress performance of the host, and (2) when endophyte and AMF infections are both present, is the effect of endophyte infection on the host’s growth and saline-alkali tolerance influenced by the AMF?

## Materials and Methods

### Plants and Fungi

Endophyte-infected (E+) tall fescue seeds were naturally infected with *Epichloë coenophialum* (Morgan-Jones and Gams, 1982; Leuchtmann et al., 2014), and uninfected (E–) seeds were acquired by eliminating the endophyte by long-term storage of E+seeds at room temperature. This procedure reduces the viability of the endophyte but not the seeds (Clay and Holah, 1990). The seeds used in this experiment were several generations removed from the storage treatment and came from freely cross-pollinated field-grown parents. The E+ and E– seeds were provided by Professor Anzhi Ren at Nankai University. The E+ and E– seeds were sown in 160 pots (80 pots for E+ and 80 pots for E– plants), with 20 seeds per pot, in sterilized vermiculite. After germination, the seedlings were thinned to 15 per pot (21 cm in diameter × 16 cm in height). The endophyte status of the plants was checked both immediately before and after the experiment by microscopic examination of leaf sheaths stained with aniline blue, as described by Latch and Christensen (1985).

FM and CE were used in this study, and these two fungi were prepared by trap culture in pots using *Sorghum bicolor* as the host plant under controlled greenhouse conditions for 3 months. The soil was then dried, and the roots were cut into < 1-cm pieces, which were subsequently homogenously mixed. The amount of inoculum for single species inoculation (FM or CE) was 100 g per pot; for the FM + CE treatment, the amount was 50 g for each AMF per pot. The M- treatment received a 100-g autoclaved inoculum and 50 ml of a non-autoclaved inoculum filtrate (passed through a 10-μm sieve) to correct for possible differences in the microbial community between the AMF and M- treatments.

### Experimental Design

The experiment was conducted from October 15, 2020 to February 15, 2021 based on a fully crossed 2 × 4 × 4 factorial design. The first factor, endophyte infection status, contained two levels: E+ and E–. The second factor, AMF inoculum, contained four levels: M-, inoculation with FM, inoculation with CE, and a combination inoculum of FM + CE. The third factor, saline-alkali treatment, contained four intensities: 0, 200, 400, and 600 mM. Four salts, NaCl, Na_2_SO_4_, NaHCO_3_, andNa_2_CO_3_, were mixed in 9:1:1:9 M ratios to simulate a range of mixed saline-alkali stress conditions. There were 32 treatment combinations in total, and each was repeated five times. During the first 6 weeks, tall fescue seedlings were grown free of saline-alkali to ensure functional mycorrhiza and to avoid the effects of saline-alkali stress on fungi development (Feng et al., 2002). The seedlings were treated with modified 1/2 strength Hoagland nutrient solution supplemented with additional 0–, 200–, 400–, and 600-mM, as well as with 50 mM saline-alkali on the first and second days to avoid saline-alkali shock. Final concentrations of saline-alkali in each treatment were applied from the third day onward. Every 3 days, the treatment solution was replaced to maintain consistent stress conditions, and the position of each pot was changed randomly.

### Harvest and Measurements

After 8 weeks of saline-alkali treatment, the shoots and roots were separated and harvested. Each pot’s roots were washed and divided into two sub-samples. AMF colonization was measured using a sample of approximately 2 g that was cleared in 10% KOH and stained in 1% trypan blue (Phillips and Hayman, 1970). AMF colonization rate was recorded using the cross-hair eyepiece method under a dissecting microscope at 40 × magnification (McGonigle et al., 1990). The remainder of the sample was used to calculate biomass and other parameters. The biomass of the plants was determined after roots and shoots were oven dried at 80°C for 24 h. The plants were ground and digested at a high temperature in a PerkinElmer microwave using an HNO_3_:HCl mixture (9:1). Phosphorus concentration was measured using acid-dissolved molybdenum, antimony, and scandium colorimetry (Chen et al., 2017). The concentrations of Na^+^ and K^+^ were assessed using ICP-OES in an Optima 7000 DV spectrophotometer (PerkinElmer, United States). Plant organic carbon (C) concentration in the shoots and roots was measured using the K_2_CrO_7_-H_2_SO_4_ oxidation method (Tanveer et al., 2014). Total nitrogen (N) was determined using a dry combustion method and an elemental analyzer (Vario EL/micro cube, Elementar, Hanau, Germany; Zhou et al., 2016).

### Statistical Analyses

The effects of *Epichloë* endophytes, AMFs, and saline-alkali stress on AMF colonization rate, plant biomass, nutrient concentration parameters (C, N, and P), and cations (Na^+^ and K^+^) were analyzed by three-factor ANOVA with SPSS 20.0 (SPSS Inc., Chicago, IL, United States). When a significant effect was detected, differences in means between different treatments were determined by Duncan’s multiple range tests at a probability of 0.05. A redundancy analysis (RDA) was performed using *Epichloë* endophytes, AMF treatments, and saline-alkali stress (S) as explanatory variables, with growth (biomass) and physiological (C, N, P, Na^+^, and K^+^) parameters as response variables. After a Monte Carlo permutation test with 499 permutations, statistical significance was determined by stepwise forward selection.

## Results

### Arbuscular Mycorrhizal Fungi Colonization Rate

**FIGURE 1:**
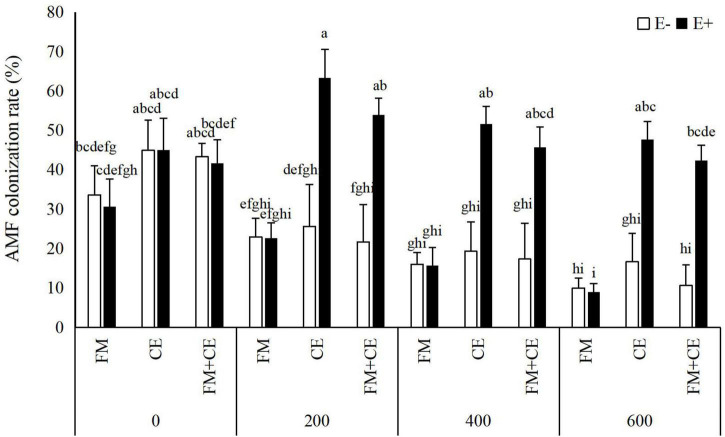
Arbuscular mycorrhizal fungi (AMF) colonization rate of tall fescue with (E+) and without (E–) endophytes and colonized with (AMF) and without (M-) AMF under saline-alkali stress. Values are means ± SE. Different letters denote means that are significantly different (*P* < 0.05).

AMF structures were not observed in the roots of M- treatments, whereas plants inoculated with AMF showed 9–63.3% root colonization rate. AMF colonization rate was significantly influenced by the *Epichloë* endophyte × saline-alkali stress interaction (Table 1). In the control treatment (0 mM), there was no difference in AMF colonization rate between E– and E+ plants, either with inoculation with FM or CE, or the mixture of FM and CE (FM + CE); however, higher colonization rate was found in E+ than in E– plants when inoculated with CE and the mixture of FM and CE (FM + CE) in the 200–, 400–, and 600-mM saline-alkali stress treatments, and no significant difference was observed between the E+ and E– plants when inoculated with FM (Figure 1).

**TABLE 1:** Three-way ANOVA for the effects of endophytes (E), arbuscular mycorrhizal fungi (AMFs, M), and saline-alkali stress (S) on AMF colonization rate, biomass, Na^+^ and K^+^ in shoots and roots of tall fescue.

	AMF colonization rate	Biomass		Na^+^		K^+^	
				
		Shoot	Root	Shoot	Root	Shoot	Root
Endophytes (E)	37.768***	30.789***	141.524***	77.542***	1667.281***	98.574***	37.151***
AMF (M)	20.784***	109.944***	217.096***	126.138***	1201.875***	219.637***	58.935***
Saline-alkali (S)	9.075***	595.509***	1080.803***	2348.554***	17194.196***	1467.438***	232.668***
E × M	10.995***	8.433***	11.416***	37.741***	205.126***	19.442***	3.913***
E × S	5.149**	11.877***	43.068***	9.015***	134.329***	13.780***	1.742^ns^
M × S	0.271^ns^	12.315***	50.434***	21.773***	195.896***	23.651***	1.877^ns^
E × M × S	1.051^ns^	2.677*	3.668**	6.323***	52.708***	1.293^ns^	1.086^ns^

The numeric data in the table is F-value. *, **, ***, and ns represent significance at the P < 5, 1, and 0.1% levels and non-significance, respectively.

### Growth Parameters

**FIGURE 2:**
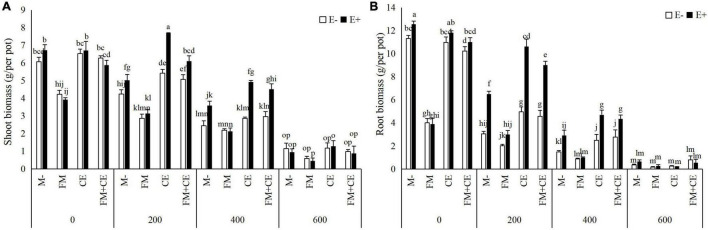
Biomass in shoots and roots of tall fescue with (E+) and without (E–) endophytes and colonized with (AMF) and without (M-) AMF under saline-alkali stress. **(A)** shoot biomass, **(B)** root biomass. Values are means ± SE. Different letters denote means that are significantly different (*P* < 0.05).

The endophytes, AMFs, and saline-alkali stress all had a significant interactive effect on shoot and root biomass (Table 1). In the control treatment (0 mM), E+ had comparable shoot and root biomass to the E– plants regardless of AMF inoculation. Under saline-alkali conditions, with the M- treatment, the shoot and root biomass of the E+ plants was greater than that of the E– plants in the 200- and 400-mM saline-alkali stress treatments. When inoculated with AMF alone, FM had a detrimental effect on shoot and root biomass, while CE was found to be beneficial to host growth in the 200- and 400-mM stress treatments. The combination of FM and CE (FM + CE) had a similar effect on host growth as the CE. The growth advantage of E+ relative to the E– plants was reduced by FM but increased by CE and those simultaneously containing the FM and CE mixture (FM + CE) in the 200- and 400-mM saline-alkali treatments. No obvious difference was observed between the E+ and E– plants in the 600-mM saline-alkali treatment regardless of AMF inoculation (Figures 2A,B).

### Carbon, Nitrogen, and Phosphorus Concentrations

**FIGURE 3:**
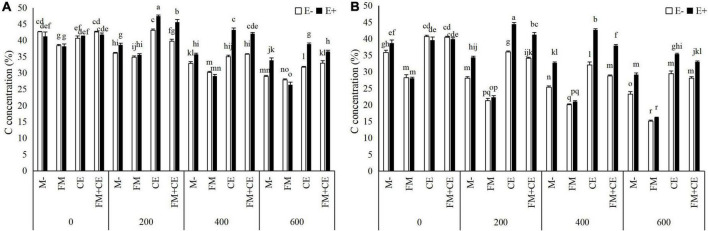
Carbon (C) concentration in shoots and roots of tall fescue with (E+) and without (E–) endophytes and colonized with (AMF) and without (M-) AMF under saline-alkali stress. **(A)** shoot C concentration, **(B)** root C concentration. Values are means ± SE. Different letters denote means that are significantly different (*P* < 0.05).

C concentration in the shoots and roots showed no significant difference between the E+ and E– plants in the control treatment (0 mM). However, in the M- treatment, C concentration under stress (200, 400, and 600 mM) conditions was higher in E+ than in the E– plants in both the shoots and roots. FM inoculation significantly decreased C concentration in the shoots and roots of both the E+ and E– plants, and there was no difference between the E+ and E– plants when inoculated with FM either in the 200–, 400–, or 600-mM saline-alkali treatment. Unlike FM, the E+ plants had a higher C concentration in the shoots and roots than the E– plants when inoculated with CE, and a synergistic effect between CE and the endophytes was observed, with C concentration in the shoots and roots of host plants simultaneously infected by CE and endophytes being significantly greater than that of plants infected with either CE or the endophytes separately. The combination of FM and CE (FM + CE) had an effect on C concentration similar to that of CE (Table 2 and Figures 3A,B).

**TABLE 2:** Three-way ANOVA for the effects of endophytes (E), AMFs (M), and saline-alkali stress (S) on C, N, and P concentrations in shoots and roots of tall fescue.

	C concentration		N concentration		P concentration	
	Shoot	Root	Shoot	Root	Shoot	Root
Endophytes (E)	203.822***	546.680***	290.541***	178.755***	818.466***	84.781***
AMF (M)	362.740***	1458.130***	425.737***	565.720***	1780.575***	336.368***
Saline-alkali (S)	488.028***	536.472***	272.580***	283.243***	15.725***	73.061***
E × M	41.748***	42.618***	16.169***	20.557***	111.896***	10.765***
E × S	31.284***	62.494***	28.369***	28.032***	46.050***	2.383^ns^
M × S	24.002***	14.596***	18.059***	56.267***	142.904***	4.155***
E × M × S	7.830***	7.876***	11.952***	5.721***	11.976***	2.306*

The numeric data in the table is F-value. *, **, *** and ns represent significance at the P < 5, 1, and 0.1% levels and non-significance, respectively.

**FIGURE 4:**
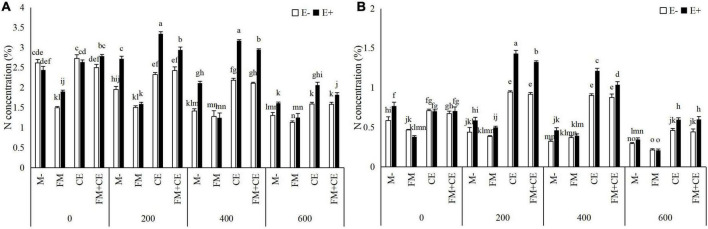
Nitrogen (N) concentration in shoots and roots of tall fescue with (E+) and without (E–) endophytes and colonized with (AMF) and without (M-) AMF under saline-alkali stress. **(A)** shoot N concentration, **(B)** root N concentration. Values are means ± SE. Different letters denote means that are significantly different (*P* < 0.05).

**FIGURE 5:**
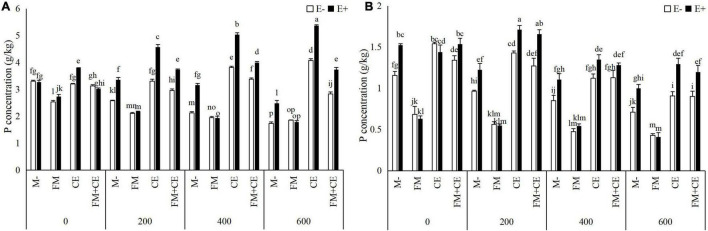
Phosphorus (P) concentration in shoots and roots of tall fescue with (E+) and without (E–) endophytes and colonized with (AMF) and without (M-) AMF under saline-alkali stress. **(A)** shoot P concentration, **(B)** root P concentration. Values are means ± SE. Different letters denote means that are significantly different (*P* < 0.05).

Saline-alkali stress significantly decreased the N and P concentration in the shoots and roots, and the decrease was significantly influenced by *Epichloë* endophyte × AMF (Table 2). In shoots and roots, the E+ plants had approximately 37 and 31% higher N concentrations, and 41 and 33% P concentrations, respectively, than the E– plants. The beneficial effects of endophytes were inhibited by the detrimental AMF, FM, and promoted by the beneficial AMF, CE, and the mixture of FM and CE (FM + CE) in the stress treatments (Figures 4 and 5).

### Na^+^ and K^+^ Concentration

The results showed that as the stress concentrations increased, the Na^+^ concentration in the shoots and roots of tall fescue increased. However, *Epichloë* endophyte presence alleviated this change, with the E+ plants having significantly lower Na^+^ concentrations in the shoots and roots than the E– plants in the M- treatment. FM inoculation increased Na^+^ concentration when compared to the M- treatment, especially in the roots. CE inoculation decreased Na^+^ concentration when compared to the M- treatment. The mixture of FM and CE (FM + CE) had an effect similar to that of CE. In addition, there was a significant interaction between the endophytes and AMF species identity on Na^+^ concentration, with the E+ plants having higher shoot and root Na^+^ concentrations than the E– plants when inoculated with FM but lower shoot and root Na^+^ concentrations when inoculated with CE and that containing both FM and CE mixture (FM + CE; Tables 1 and 3).

**TABLE 3:** Na^+^ and K^+^ concentrations in shoots and roots of tall fescue with (E+) and without (E–) endophytes and colonized with (AMF) and without (M-) AMF under saline-alkali stress (mean ± SE, *n* = 3).

		Na^+^				K^+^			
		Shoot		Root		Shoot		Root	
		E–	E+	E–	E+	E–	E+	E–	E+
0	M-	0.19 ± 0.01m	0.20 ± 0.01m	0.22 ± 0.01r	0.19 ± 0.01st	3.37 ± 0.11ab	3.39 ± 0.10ab	1.73 ± 0.08cd	1.79 ± 0.03bc
	FM	0.16 ± 0.01m	0.17 ± 0.01m	0.18 ± 0.01st	0.18 ± 0.01t	3.21 ± 0.01bcd	3.11 ± 0.00de	1.48 ± 0.05efgh	1.57 ± 0.06defg
	CE	0.22 ± 0.01m	0.17 ± 0.00m	0.20 ± 0.01rs	0.15 ± 0.01u	3.31 ± 0.02abc	3.42 ± 0.07a	2.09 ± 0.03a	2.03 ± 0.04a
	FM + CE	0.14 ± 0.02m	0.15 ± 0.02m	0.15 ± 0.01u	0.15 ± 0.01u	3.27 ± 0.05abcd	3.16 ± 0.09cd	1.98 ± 0.07ab	2.09 ± 0.06a
200	M-	0.95 ± 0.05ij	0.83 ± 0.01jk	0.75 ± 0.01j	0.53 ± 0.00n	2.04 ± 0.05hij	2.49 ± 0.02f	1.31 ± 0.15hij	1.57 ± 0.12defg
	FM	1.08 ± 0.04hi	1.18 ± 0.04gh	0.82 ± 0.01hi	0.85 ± 0.01g	2.05 ± 0.01hij	1.96 ± 0.07ij	1.26 ± 0.06hijkl	1.26 ± 0.05hijkl
	CE	1.00 ± 0.07i	0.63 ± 0.08l	0.56 ± 0.00n	0.40 ± 0.01q	2.49 ± 0.09f	2.95 ± 0.02e	1.55 ± 0.07def	1.81 ± 0.05bc
	FM + CE	1.07 ± 0.04hi	0.75 ± 0.02k	0.62 ± 0.01l	0.42 ± 0.00pq	2.23 ± 0.06gh	2.57 ± 0.04f	1.42 ± 0.05fgh	1.65 ± 0.07cde
400	M-	1.34 ± 0.06f	0.99 ± 0.09i	0.92 ± 0.01e	0.62 ± 0.01l	1.51 ± 0.03kl	1.89 ± 0.03j	1.17 ± 0.10ijklm	1.31 ± 0.05hij
	FM	1.26 ± 0.00fg	1.64 ± 0.05d	0.83 ± 0.00ghi	0.81 ± 0.00i	1.34 ± 0.12lm	1.29 ± 0.05m	1.11 ± 0.04jklmn	1.07 ± 0.05klmno
	CE	1.07 ± 0.03hi	0.77 ± 0.03k	0.60 ± 0.00m	0.44 ± 0.00p	2.15 ± 0.04h	2.55 ± 0.04f	1.37 ± 0.10ghi	1.63 ± 0.07cdef
	FM + CE	1.14 ± 0.05gh	0.85 ± 0.03jk	0.70 ± 0.01k	0.51 ± 0.00o	2.12 ± 0.06hi	2.39 ± 0.04fg	1.14 ± 0.10jklm	1.42 ± 0.09fgh
600	M-	2.01 ± 0.02b	1.75 ± 0.02cd	1.19 ± 0.03a	1.15 ± 0.01b	1.10 ± 0.06n	1.54 ± 0.07k	0.89 ± 0.01op	1.07 ± 0.09lmno
	FM	2.31 ± 0.03a	2.36 ± 0.10a	1.09 ± 0.01c	1.10 ± 0.01c	0.81 ± 0.03o	0.82 ± 0.05o	0.82 ± 0.01p	0.79 ± 0.01p
	CE	1.78 ± 0.02c	1.46 ± 0.03e	1.05 ± 0.01d	0.84 ± 0.01gh	1.52 ± 0.06kl	1.95 ± 0.02ij	0.99 ± 0.01mnop	1.29 ± 0.01hijk
	FM + CE	1.95 ± 0.05b	1.68 ± 0.05cd	1.17 ± 0.01b	0.89 ± 0.01f	1.46 ± 0.04klm	1.86 ± 0.09j	0.92 ± 0.04nop	1.16 ± 0.05ijklm

A decrease in K^+^ concentration was observed in the shoots and roots as the stress concentrations increased. However, the presence of the endophytes alleviated the decrease, particularly in shoot K^+^ concentration. In the M- treatment, the E+ plants had significantly higher K^+^ concentrations in the shoots in the 200–, 400–, and 600-mM saline-alkali treatments, as well as a higher K^+^ concentration in the roots in the 200-mM saline-alkali treatments. FM inoculation had no significant effect on K^+^ concentration in shoots and roots of the E– plants but significantly decreased K^+^ concentration in shoots and roots of THE E+ plants, resulting in disappearance of the E+ advantage over the E– plants. Under all stress conditions, a synergistic effect occurred between CE and the endophytes, and K^+^ concentration in the shoots and roots of host plants simultaneously infected by CE and the endophytes was significantly greater than that of plants infected with either CE or the endophytes separately (Tables 1 and 3).

### Redundancy Analysis

**FIGURE 6:**
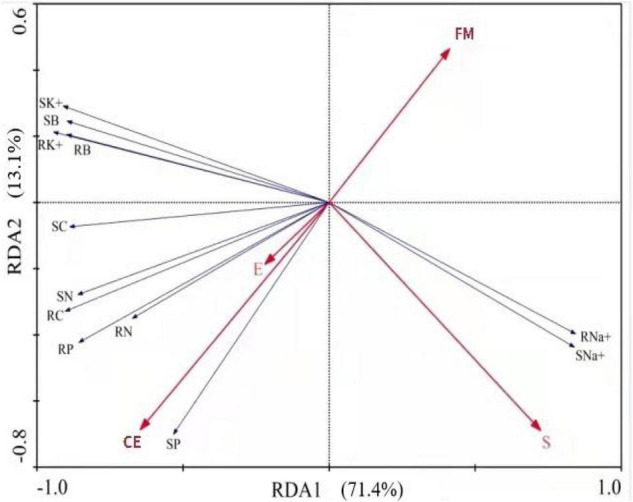
Redundancy analysis (RDA) of *Epichloë* endophytes (E), AMFs (FM, *Funneliformis mosseae* and CE, *Claroideoglomus etunicatum*) and saline-alkali stress (S) on growth and physiological parameters of tall fescue under saline-alkali stress. Shoot biomass (SB), root biomass (RB), shoot C (SC), root C (RC), shoot N (SN), root N (RN), shoot P (SP), root P (RP), shoot Na^+^ (SNa^+^), root Na^+^ (RNa^+^), shoot K^+^ (SK^+^), root K^+^ (RK^+^).

Three factors, *Epichloë* endophytes, AMFs, and saline-alkali stress (S), as well as growth and physiological parameters of host plants, biomass, nutrients (C, N, and P), and cation (Na^+^ and K^+^) concentration, were used for RDA to investigate the contributions of both the endophytes and the AMFs to the growth of tall fescue under saline-alkali stress conditions. Axis 1 of the RDA plot explained 71.4% of the total variance by being positively correlated with endophytes and CE and negatively correlated with S and FM. Axis 2 explained 13.1% of the total variance. The endophytes, FM, CE, and S explained 4, 15, 36, and 44% of the total variance, respectively (Figure 6).

## Discussion

Many studies have reported the *Epichloë* endophytes’ ability to increase host resistance to abiotic stresses (Wang J. F. et al., 2020; Wang Z. F. et al., 2020; Chen et al., 2021; Liu et al., 2021; Wang et al., 2021). Among abiotic stress conditions, we demonstrated that *Epichloë-*infected tall fescue outperformed uninfected plants in saline-alkali soil. The ability of *Epichloë* to improve resistance to NaCl stress, a single physiological stress, has been demonstrated in many studies (Chen S. et al., 2018; Chen T. X. et al., 2018, 2019; Wang et al., 2019; Chen et al., 2021); however, very little is known about how *Epichloë* infection impacts mixed saline-alkali stress. The results of our study, first, revealed that endophyte presence resulted in higher biomass of shoots and roots under saline-alkali stress (200 and 400 mM) relative to that of the E– plants, which was consistent with previous research results (Chen T. X. et al., 2018, 2019; Wang et al., 2019). Second, *Epichloë* endophyte infection significantly decreased the concentration of Na^+^ and increased the concentration of K^+^ under saline-alkali conditions, which was consistent with the findings of Song et al. (2015). The reduction of Na^+^ due to endophytes can alleviate the damage of plant cells by decreasing the inhibition of enzymes, disrupting K^+^ acquisition, and inhibiting K^+^-dependent metabolic processes on the one hand, and reducing oxidative stress on the other (Chen T. X. et al., 2018; Chen T. X. et al., 2019; Chen J. B. et al., 2019). Under saline-alkali stress, K^+^ accumulation by endophytes is important for stomatal conductance and maintenance of normal plant activities (Chen J. B. et al., 2019). *Epichloë* endophytes regulate the balance of Na^+^ and K^+^ in plants, thus maintaining normal metabolic processes in cells and improving the adaptation of plants to saline-alkali environments (Song et al., 2015; Chen S. et al., 2018; Wang et al., 2021). Third, the E+ plants had higher concentrations of C, N, and P in the shoots and roots than the E– plants under saline-alkali stress conditions, which can improve plant metabolism by promoting protein synthesis and increasing the concentration of compatible osmolytes, as well as helping to maintain cell membrane integrity and reducing electrolyte leakage (Song et al., 2015; Chen T. X. et al., 2018; Wang et al., 2021).

Both *Epichloë* and AMF metabolize carbohydrates from host plants. Previous studies have produced inconsistent results when *Epichloë* and AMF simultaneously interact with a host, reporting that AMF root colonization rate was inhibited (Omacini et al., 2006; Liu et al., 2011) or promoted (Novas et al., 2005; Vignale et al., 2016) by *Epichloë* endophyte infection. In our study, we demonstrated that *Epichloë* and saline-alkali stress had significant interactive effects on AMF colonization rate. Under the control treatment, *Epichloë* endophyte infection had no significant effects on AMF colonization rate regardless of AMF species. Under the saline-alkali stress treatment, the presence of *Epichloë* endophytes significantly decreased the colonization of FM but increased the colonization rate of CE and the mixture of FM and CE (FM + CE). These discrepancies of *Epichloë* endophytes in AMF colonization may be attributed to AMF species (Larimer et al., 2012; Zhou et al., 2016) and environmental conditions (Li et al., 2019). Larimer et al. (2012) showed that *Epichloë elymi* significantly promoted the colonization rate of FM but inhibited the colonization rate of *Claroideoglomus claroideum* (CC). Zhou et al. (2016) found that *Epichloë* endophyte infection significantly reduced the colonization rate of CE but did not affect the colonization rate of FM. Li et al. (2019) demonstrated that the effect of *Epichloë* endophyte infection on the colonization rate of CE was influenced by soil water content. Endophyte infection significantly reduced the colonization rate of CE at 70% soil water content but had no effect on that of CE at either 50 or 30% soil water content.

When tripartite interactions among *Epichloë* endophytes, AMFs, and host plants were considered, some studies found that the effects of *Epichloë* endophyte infection on host plants were influenced by AMFs (Zhou et al., 2016; Liu et al., 2017, 2019; Li et al., 2019). For example, Zhou et al. (2016) reported that the effects of *Epichloë* endophyte infection on the shoot biomass of *A. sibiricum* changed from neutral with the M- treatment to positive with the FM inoculation treatment under sufficient N and P conditions. Li et al. (2019) indicated that there was no significant effect of *Epichloë* endophyte infection on the total P content of *Lolium perenne* with the M- treatment but significantly increased total P content with AMF inoculation treatment under 70% soil water content conditions. Liu et al. (2019) discovered that the outcomes of tripartite interactions among *Epichloë* endophytes, AMFs, and host plants varied with AMF identity. The beneficial effect of endophyte infection on plant shoot biomass decreased in response to FM but increased in response to *Rhizophagus intraradices* (RI). No obvious difference was observed between the E+ and E–plants when inoculated either with CE or CC. Some studies, however, reported no interaction between *Epichloë* endophytes and AMFs in host plants (Omacini et al., 2006; Mack and Rudgers, 2008; Larimer et al., 2012). In our study, we found that the interaction between *Epichloë* endophytes and AMFs has a significant influence on the saline-alkali resistance of tall fescue, and that this was dependent on the species of AMFs. For the E– plants, host plant resistance to saline-alkali stress was decreased by FM but increased by CE. FM inoculation significantly decreased plant biomass (shoot and root biomass), nutrient concentrations (C, N, and P), and K^+^ concentration while increasing Na^+^ concentration. Contrary to FM, CE inoculation significantly increased these response variables. However, there was an antagonistic interaction between the *Epichloë* endophytes and the detrimental AMF, FM, but a synergistic interaction between the *Epichloë* endophytes and the beneficial AMF, CE, in plant biomass (shoot and root biomass), nutrient concentrations (C, N, and P), and ion concentration (Na^+^ and K^+^) for the E+ plants. Thus, the differences between the E+ and E– plants under saline-alkali stress conditions were reduced by FM but increased by CE.

According to the RDA analysis, the contribution of endophytes was less than that of AMFs and further suggested that the AMF species played an important role in *Epichloë* endophyte-host-AMF tripartite interactions. This result was similar to the findings of Zhou et al. (2016) who found that the contribution of endophytes to *A. sibiricum* was less than that of AMFs under conditions of nutrient stress. AMFs were present in the roots of host plants, and they directly absorbed N and P *via* external AMF hyphae in soil (Kong et al., 2019), whereas *Epichloë* endophytes lived in the above-ground tissues of the host plants (Wang J. F. et al., 2020) and indirectly affected nutrient absorption by changing the root’s morphological and physiological characteristics (Chen et al., 2020, 2021). This may be the reason that could explain why *Epichloë* endophytes have lower contribution than AMFs under saline-alkali stress conditions.

## Conclusion

In conclusion, a significant interaction among the *Epichloë* endophytes, AMFs, and saline-alkali stress occurred in tall fescue growth and physiological parameters. Endophyte infection significantly enhanced tall fescue resistance to saline-alkali stress by increasing biomass, nutrient uptake, and accumulation of K^+^ while decreasing Na^+^ concentration; this beneficial effect of the endophytes was enhanced by the beneficial AMF, CE, but reduced by the detrimental AMF, FM. Our study reinforces the currently limited finding that *Epichloë* endophytes and AMFs interact in complex ways to influence the growth of their shared host grasses, especially under stress conditions. Further research should be conducted to investigate the ecological implications of the combined effects of *Epichloë* endophytes and AMFs under field conditions.

## Data Availability Statement

The original contributions presented in the study are included in the article/supplementary material, further inquiries can be directed to the corresponding author.

## Author Contributions

HL designed the research and revised and polished the manuscript. HT, XN, YZ, and YW performed the experiments. HL and HT analyzed the data and wrote the manuscript. All authors contributed to the article and approved the submitted version.

## Conflict of Interest

The authors declare that the research was conducted in the absence of any commercial or financial relationships that could be construed as a potential conflict of interest.

## Publisher’s Note

All claims expressed in this article are solely those of the authors and do not necessarily represent those of their affiliated organizations, or those of the publisher, the editors and the reviewers. Any product that may be evaluated in this article, or claim that may be made by its manufacturer, is not guaranteed or endorsed by the publisher.
